# Monitoring measles infections using flight passenger dynamics in Europe: A data-driven approach

**DOI:** 10.1038/s41597-024-04231-x

**Published:** 2024-12-18

**Authors:** Chiara Romano, Francesco Branda, Fabio Scarpa, Giovanna Jona Lasinio, Massimo Ciccozzi

**Affiliations:** 1 Unit of Medical Statistics and Molecular Epidemiology, Università Campus Bio-Medico di Roma, 00128 Rome, Italy; 2 Department of Biomedical Sciences, University of Sassari, 07100 Sassari, Italy; 3 Department of Statistical Sciences, Sapienza University of Rome, 00185 Rome, Italy

**Keywords:** Infectious diseases, Databases

## Abstract

This paper presents an open-access repository collecting information on measles virus infections and flight passenger movements in European countries from 2011 to 2023. It provides a comprehensive overview of reported measles cases and measles-mumps-rubella (MMR) vaccination coverage from authoritative organizations such as the World Health Organization (WHO) and the European Centre for Disease Prevention and Control (ECDC). In addition, the dataset includes detailed data on passenger movements between countries, facilitating analysis of cross-border disease transmission. This resource enables more precise spatial analyses for monitoring and forecasting measles outbreaks, underscoring the importance of adequate vaccination coverage and sustained international surveillance to prevent the spread of the disease.

## Background & Summary

Measles, caused by *Morbillivirus*, a member of the Paramyxoviridae family, represents one of the most contagious viral diseases known to humans. Measles virus (MV) has a negative-sense single-stranded RNA genome and is mainly transmitted by airborne route through respiratory droplets or direct contact with infected secretions^1^. MV has an incubation period of about 10–12 days, during which the patient is asymptomatic but already contagious^2^. The disease begins with general symptoms such as high fever, cough, rhinitis, and conjunctivitis. After a few days, the typical rash appears, consisting of red macules extending from the face to the rest of the body. One of the distinctive clinical signs is the appearance of Koplik’s spots, small whitish lesions inside the mouth^3^. Measles is highly contagious: it is estimated that an infected person can transmit the virus to about 90% of nonimmune persons with whom he or she comes in contact^4^. This contagiousness is compounded by the ability of the virus to remain active in the air or on surfaces for several hours, thus increasing the likelihood of transmission^5^. Measles can have serious complications, especially in malnourished children and people with compromised immune systems. Common complications include encephalitis, which can lead to permanent brain damage, pneumonia, and blindness^6^. In developing countries, where malnutrition and limited access to health services are prevalent, the mortality rate associated with measles can be as high as 10–15%^7^. Even in industrialized countries, measles can cause severe complications in unvaccinated adults and in infants too young to receive the vaccine. One of the most worrisome consequences of measles infection is the transient immunosuppression that the virus induces. After an infection, the immune system can remain weakened for several months, making the individual susceptible to other infections^8^. Recent studies have shown that measles can “reset” immune memory, leading to a loss of acquired immunity against other diseases, a phenomenon known as “immune amnesia”^6^.

The introduction of the measles vaccine combined with mumps and rubella (MMR) has dramatically reduced global mortality and morbidity, especially among children^9^. Between 2000 and 2016, there was a 79% reduction in measles-related deaths due to increased vaccination coverage, which saved about 20 million lives^10^. However, in recent years, measles has experienced a resurgence in several regions of the world, including Europe, with numerous outbreaks in Italy, France, and Germany^11^. In 2019, the number of global cases increased by 300% from the previous year, with significant outbreaks in low- and middle-income countries^12^. The surge in cases in recent years can be attributed to several factors, including the spread of misleading information regarding the safety of vaccines, which has led to a decline in confidence in vaccination in some communities^13^. In addition, socioeconomic inequalities within and between countries have created significant disparities in access to vaccination^14^, putting vulnerable populations at greater risk of infection. Measles prevention, therefore, continues to depend largely on the implementation of extensive vaccination programs and the ability to maintain high rates of vaccination coverage.

However, to ensure global control of the disease, constant monitoring and open access to measles epidemiological data could be a significant step forward for research and prevention. Open data would allow researchers to analyze the spread of the virus in greater depth, for example by mapping gaps in vaccination coverage and identifying areas at high risk of transmission, predicting outbreaks, and improving immunization strategies^15–17^. Publicly accessible datasets also foster collaboration between health institutions, researchers, and policymakers, enhancing the capacity to respond swiftly to future outbreaks. Such collaboration leads to greater transparency in managing outbreaks and enables shared decision-making based on real-time data, which is critical for timely public health responses. This cooperative framework strengthens global efforts to contain infectious diseases by integrating diverse expertise and resources into a unified approach^18^.

In this context, the paper presents an open-access repository that includes data on (i) observed measles virus cases, annual coverage of first and second doses of MMR vaccine, and annual population in 32 European countries from 2011 to 2023; and (ii) total number of air passengers between each pair of countries in the years 2019, 2020, and 2022. With a broad temporal and geographic coverage, the repository provides a detailed and continuous overview of measles dynamics in Europe, facilitating monitoring of changes over time and cross-country comparisons. The integration of data on measles cases, vaccination coverage, and demographic characteristics provides a solid basis for examining how the spread of the virus interacts with vaccination effectiveness and population characteristics. In addition, data on airline passengers allow analysis of how international movements influence the spread of measles, helping to identify routes at greatest risk and assess the impact of travel and quarantine policies. This is particularly useful for developing prevention and response strategies based on a thorough understanding of global transmission dynamics. Open access to this dataset promotes transparency and collaboration among researchers, health institutions, and policy makers, improving the ability to formulate informed and timely health policies. In sum, the dataset is a valuable tool for epidemiological analysis, health policy planning, and evaluation of the effectiveness of vaccination campaigns, contributing to more effective management of public health emergencies.

The remainder of the paper is organized as follows: the Methods section details the data sources used and the procedures adopted to create the final dataset. Next, the Data Records section provides an in-depth presentation of the dataset, explaining the structure and content of the data collected. In the Technical Validation section, assessments are made to ensure the consistency and statistical reliability of the data, highlighting any limitations and how they were addressed. Finally, in the Usage Notes section, the potential of the dataset for future research purposes is discussed, exploring possible practical applications and how it may contribute to the development of new study and prevention strategies.

## Methods

Data were collected from 32 European countries, comprising the 27 member states of the European Union: Austria, Belgium, Bulgaria, Croatia, Cyprus, Czech Republic, Denmark, Estonia, Finland, France, Germany, Greece, Hungary, Ireland, Italy, Latvia, Lithuania, Luxembourg, Malta, the Netherlands, Poland, Portugal, Romania, Slovakia, Slovenia, Spain, and Sweden. Additionally, Montenegro, Norway, Serbia, Switzerland, and the United Kingdom were included. The dataset spans a 13-year period, from 2011 to 2023, and was assembled using public sources such as Eurostat, the World Health Organization (WHO), and the European Centre for Disease Prevention and Control (ECDC). The first dataset created is called “measles.xlsx” and includes the number of observed measles cases, the annual coverage of the first and second doses of the MMR vaccine, and the total population of the European countries from 2011 to 2023, each of which is described in detail in the following: (i) number of measles cases observed for the EU/EEA countries were downloaded from The Surveillance Atlas of Infectious Diseases, an ECDC tool that provides monthly measles cases from January 2011 to December 2023 for all countries of interest^19^ (ECDC_measles_cases). The dataset provided by ECDC is based on data provided by WHO and Ministries of Health from the affected countries. For the five countries Montenegro, Norway, Serbia, Switzerland, and the United Kingdom the reported number of measles cases were downloaded from the World Health Organization data repository^20^ (WHO_measles_cases). The indicator between the two data providers is the same. The data have not been transformed and have no missing information. (ii) Regional data on vaccination coverage come from WHO, specifically the WHO/UNICEF Joint Estimates of National Vaccination Coverage (WUENIC), updated to June 26, 2023^21^ (WHO_MMRcoverage). These data estimate the percentage of children who have received the first and second doses of the MMR vaccine. Missing data, especially in the second dose of MCV, were left blank. (iii) Population data were downloaded from Eurostat website and customized to include only the information of interest for the 32 countries from 2011 to 2023^22^ (Eurostat_population). Missing data for the United Kingdom population from 2021 to 2023 were completed using the 2022 Revision of World Population Prospects, the twenty-seventh edition of the official United Nations population estimates^23^ (UnitedNation_pop). The three files were ultimately merged using the left_join function from the R package *dplyr*, with the three-letter country codes (ISO 3166-1 alpha-3) and the year variable serving as the key fields for the join. This operation maintains all rows of the main dataset, the one with monthly measle’s reported cases, and repeats the annual population and vaccination coverage values for each month of the corresponding year.

The creation of the air passenger dataset aims to quantify flows of people between countries, a key element in modeling and monitoring epidemics, as population movement affects the spread of infectious diseases. Data on air routes were collected from the Eurostat website^24^ for each country, while data provided by the UK Civil Aviation Authority website^25^ were used for routes between the UK and other countries in 2020^26^ (UKCCA_flights2020) and 2022^27^ (UKCCA_flights2022). The downloaded files were processed using a custom R function, named “Route_num”, which was applied to each country and year to calculate the total number of passengers traveling between countries.

## Data Records

**Fig. 1:**
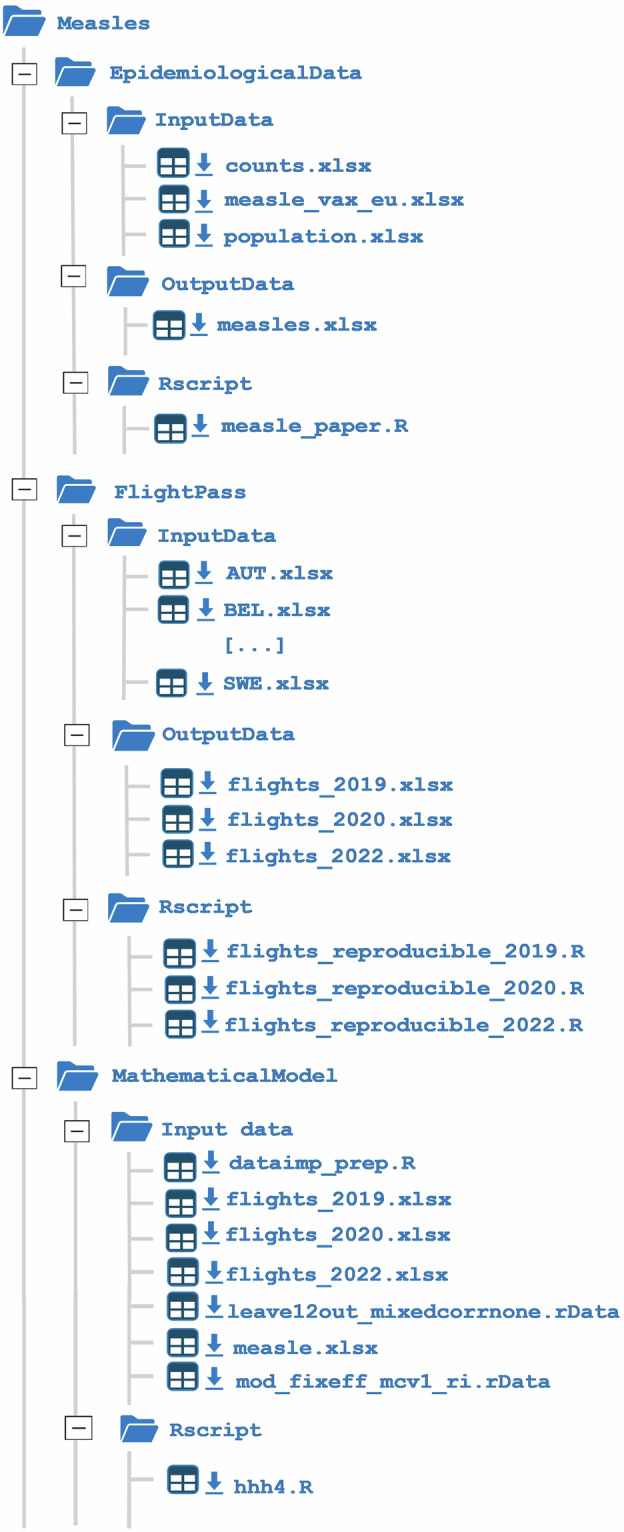
Schematic structure of the dataset.

The peer-reviewed data presented in this paper corresponds to version 2 (V2) of the dataset, which has been deposited on Zenodo^28^. The final dataset contains 4992 observations, corresponding to the product of 12 months, 13 years, and 32 countries, ranging from 2011 to 2023. The repository is organized into three distinct sections: “EpidemiologicalData”, containing the related measles information; “FlightPass”, which includes flight trajectory and passenger data used for analysis; and “MathematicalModel”, comprising the computational models developed to simulate and predict the progression of the outbreak. The structure of the repository is outlined in Fig. 1. Each of these sections consists of three folders: “InputData”, which contains the input data; “OutputData”, which collects the analysis results; and “RScript”, which includes the R scripts used for processing and modeling. A detailed description of the file structure is provided below.

### EpidemiologicalData

The input datasets located in the “InputData” folder consist of the following files: “counts.xlsx” (i.e., data on the number of observed measles cases), “measle_vax_eu.xlsx” (i.e., coverage of the first and second doses of the MMR vaccine), and “population.xlsx” (i.e., total population across 32 countries from 2011 to 2023), thoroughly described in the Methods section of this paper. The “Rscript” folder contains the script “measle_paper.R”, which was used to merge the input datasets. The structure of the resulting dataset, named “measles.xlsx”, is summarized in Table 1.

**Table 1:** Structure of the file “measles.xlsx” within the folder “EpidemiologicalData”.

Variable	Description	Format
HealthTopic	The health-related topic of the dataset (e.g., Measles).	String
Indicator	The type of health measure reported (e.g., Reported cases).	String
Time	The specific time period for the reported data (e.g., year and month).	YYYY-MM
Alpha3	Three-letter ISO country code (e.g., AUT for Austria).	String
RegionName	The name of the region or country (e.g., Austria).	String
Counts	Number of reported cases of the health topic.	Numeric
Year	The year of the reported data (e.g., 2011).	YYYY
Alpha2	Two-letter ISO country code (e.g., AT for Austria).	String
Pop_tot	Total population of the country.	Numeric
Measle_coverage	Source of vaccination coverage data (e.g., WUENIC).	String
MCV1	First-dose measles vaccination coverage in percentage.	Numeric
MCV2	Second-dose measles vaccination coverage in percentage.	Numeric

### FlightPass

**Fig. 2:**
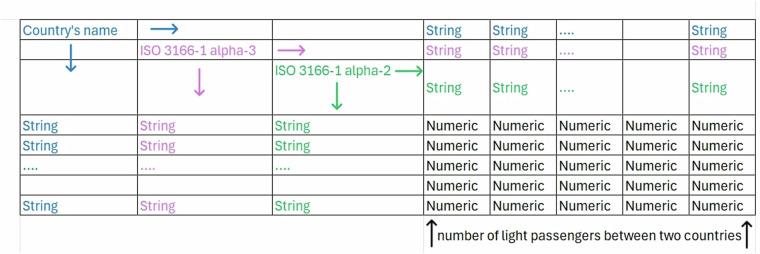
Structure of the file “flights_yyyy.xlsx” within the folder “FlightPass”.

The input dataset are Excel files named “XXX.xlsx”, where “XXX” is the three-letters country’s code (ISO 3166-1 alpha-3). The only exception are the files “Eng_2020.csv” and “Eng_2022.csv” which are the files collecting the raw data for the United Kingdom in 2020 and 2022. Finally, the datasets “GBR_2020_paper.xlsx” and “GBR_2022_paper.xlsx” are a version of the 2020 and 2022 United Kingdom’s routes data that looks like the other countries’ files. The file “flights_E.xlsx” is an empty 32 × 32 matrix used as a starting point to get the files in the folder “OutputData”. The folder “Rscript” contains the files “flights_reproducible_2019.R”, “flights_reproducible_2020.R” and “flights_reproducible_2022.R” which was used to generate the datasets “flights_2019.xlsx”, “flights_2020.xlsx”, “flights_2022.xlsx”. i.e., air travel data for 2019, 2020, and 2022, respectively, covering the pre-pandemic, pandemic, and post-pandemic periods. Each dataset is organized as a 32 × 32 matrix (see Fig. 2), where the rows and columns represent the countries analyzed. The matrix values indicate the number of air passengers traveling between each country pair, with diagonal entries showing domestic flights within each country. Note that the matrices are asymmetrical, and missing route data are left blank.

### MathematicalModel

The “InputData” folder within the “MathematicalModel” section contains the essential files for running the Held, Höhle, Hofmann (HHH) model, namely “hhh4.R”, to reproduce the results presented in this work. The folder contains necessary input data files, such as “flights_2019.xlsx”, “flights_2020.xlsx”, “flights_2022.xlsx”, and “measles.xlsx”. In addition, it includes utility files such as “dataimp_prep.R”, which prepares data for modeling and analysis of measles spread and vaccination effectiveness in relation to population changes and international travel, as well as “leave12out_mixedcorrnone.rData” and “leave12out_mixedcorrnone.rData” to speed up model execution. The former stores all the estimated models for each possibile formulation of the HHH model. The latter contains all the one-step-ahead prediction, for each model formulation, used to construct the Root Mean Square Error (RMSE). By using the HHH script, users can not only replicate the outcomes but also delve into the underlying model dynamics, which are based on multi-year flight and epidemiological data. This approach ensures both transparency and rigor in validating the study’s findings.

## Technical Validation

The data used in this study are affected by the availability and quality of source sources, with some specific issues related to data collection for the United Kingdom (UK) in 2020. In particular, UK air route and population information comes from different databases than those used for other countries, which may introduce differences in collection methodologies or estimation. However, it is important to note that all data sources used for this study are Official Statistics, which adhere to strict standards defined by international regulations and protocols. To further ensure the robustness of the data integration process, we performed manual validation by selecting successive samples of 25 observations from the measles dataset and cross-referencing each with the original data source for consistency and coherence. In the “measles” dataset, there are some missing values for the variable “MCV2”, which represents the estimated coverage of the second dose of measles vaccine. These gaps are due to incomplete information from the underlying data sources. Similarly, in the “FlightPass” dataset, the missing values are attributed to the absence of direct air routes between some countries. To validate the integrity of the compiled datasets, we applied the HHH model, a multivariate time series model for infectious disease counts, a description of which is given below.

### Held, höhle & hofmann model

The HHH model^29^ is a multivariate count data time series model for routine surveillance data, which does not require the number of susceptibles available. The observed number of measles cases (variable “counts” in “measles” dataset, see Table 1) are assumed to follow a negative binomial:

1 $${Y}_{t,k} \sim NB({\mu }_{t,k},{\psi }_{k}),\,t=\mathrm{2,}\ldots ,T,\,k=\mathrm{1,}\ldots ,K$$

2 $${\mu }_{t,k}={\nu }_{t,k}+{\lambda }_{t,k}{Y}_{t-\mathrm{1,}k}+{\phi }_{t,k}\sum _{j\ne k}{w}_{j,k}{Y}_{t-\mathrm{1,}j}$$

where, in our case, time *t* goes from January 2011 to December 2023 and *k* = 32 represent the analyzed European countries. The measles incidence *μ*_*t,k*_ can be decomposed additively into two parts: (i) the epidemic component, ${\lambda }_{t,k}{Y}_{t-\mathrm{1,}k}+{\phi }_{t,k}{\sum }_{j\ne k}{w}_{j,k}{Y}_{t-\mathrm{1,}j}$, should be able to capture occasional outbreaks whereas (ii) the endemic component, *v*_*t,k*_, explains a baseline rate of cases that is persistent with a stable temporal pattern. *v*_*t,k*_, *λ*_*t,k*_ and *ϕ*_*t,k*_ are linear predictors covering, respectively, the endemic component as well as the within- and between-unit autoregressive behaviour:

3 $$log({\nu }_{t,k})={\alpha }^{(\nu )}+{b}_{k}^{(\nu )}+{c}_{t}^{(\nu )}+{{\rm{offset}}}_{t,k}+{z}_{t,k}^{(\nu ){\prime} }{\beta }^{(\nu )}$$

4 $$log({\lambda }_{t,k})={\alpha }^{(\lambda )}+{b}_{k}^{(\lambda )}+{z}_{t,k}^{(\lambda ){\prime} }{\beta }^{(\lambda )}$$

5 $$log({\phi }_{t,k})={\alpha }^{(\phi )}+{b}_{k}^{(\phi )}$$

where *α*^(·)^ are component-specific intercept (also called fixed effect), ${b}_{k}^{\mathrm{(.)}}$ are spatial random effects, *β*^(·)^ the vector of coefficients for covariates ${z}_{t,k}^{\mathrm{(.)}}$ and ${c}_{t}^{(\nu )}$ the seasonal effect. In this application, the overdispersion parameter is identical in every unit: ${\psi }_{k}=\psi \forall k$. If the units are regions, as in our case, this may be a realistic assumption.

The fitting procedure is implemented in the R package “surveillance”^30^, with the function “hhh4”. This function allows for a variety of combinations of fixed, random, seasonal, and other covariate effects, as each of the three components can be modelled with different effects. To address, among more than 500 model scenarios, which is the best model to fit the data, we used the Root Mean Square Error (RMSE) as validation index:

$${\rm{RMSE}}=\sqrt{\frac{1}{12}{\mathop{\sum }\limits_{t=1}^{12}({\hat{\mu }}_{t,k}-{\mu }_{t,k})}^{2}}$$

The estimated values ${\hat{\mu }}_{t,k}$ are the last year’s predicted values (*January2023−December2023*) using all the other times as a validation set. The values *μ*_*t,k*_ are the reported monthly measles cases in 2023. The function “oneStepAhead”, computes successive one-step-ahead predictions for a (random effects) HHH model fitted by hhh4. This function will refit the model for the first time points until week 144 (December 2022), and use this specific fit to calculate all subsequent predictions (January 2023 to December 2023).

In the best model, the between-units autoregressive component, Eq. 5, consists of a fixed effect *α*^(*ϕ*)^. The neighbourhood weights are the number of flight passengers in 2019, “flights_2019.xlsx”, for *t* = 2011…2019, the number of flight passengers in 2020, “flights_2020.xlsx”, for *t* = 2020, 2021 and the number of flight passengers in 2022, “flights_2022.xlsx”, for *t* = 2022, 2023. The within-units autoregressive component, Eq. 4, consists of a fixed effect *α*^(*λ*)^, an independent and identical distributed (iid) Gaussian spatial effect ${b}_{k}^{(\lambda )}$ and the covariate “proxi_tot_cov”. The covariate “proxi_tot_cov” is a weighted sum of the coverage of both MCV vaccine doses (0.7*MCV1 + 0.3*MCV2). The endemic component consists of a fixed effect *α*^(*v*)^, the covariate “proxi_tot_cov” and a fixed seasonal effects ${c}_{t}^{(\nu )}$ of the form with ω_*s*_ = 2*sπ*/12 due to monthly data. We use the population “Pop_tot” in each country *k* and year as offset in the epidemic component.

$$\mathop{\sum }\limits_{s=1}^{4}\{{\gamma }_{s}\,\sin ({\omega }_{s}t)+{\delta }_{s}\,\cos ({\omega }_{s}t)\}$$

**Fig. 3:**
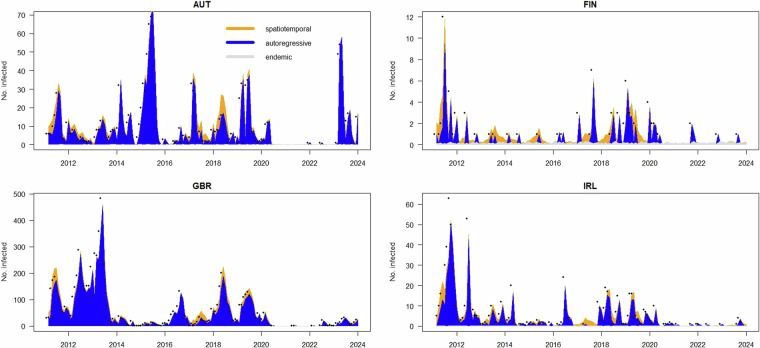
Observed cases and the hhh4 best model estimated measles trend in Austria, Finland, United Kingdom and Ireland, 2011–2023. The best model’s components are displayed in three different colors while the observed counts are black dots.

Figure 3 presents the observed measles cases alongside the estimated trends from the best-fitting hhh4 model for Austria, Finland, the United Kingdom, and Ireland, spanning the years 2011 to 2023. The model’s key components are represented in distinct colors: the between-countries epidemic component is shown in orange, the within-countries epidemic component in blue, and the endemic component in grey. Black dots represent the reported measles cases. Across all four countries, the model demonstrates a strong fit with the observed data, with the epidemic components playing a more significant role than the endemic component. Notably, in countries like Finland, which reported fewer measles cases, the between-countries epidemic component becomes more influential in capturing higher spikes in the data, compared to countries with more substantial case counts. This model can be further applied to forecast future measles trends in the countries analyzed.

## Usage Notes

This paper provides a comprehensive epidemiological overview of measles infection rates and MMR vaccination coverage in Europe, all consolidated in a single repository. In addition to epidemiological data, the collection includes information on flight passenger movements, providing valuable context for understanding the spread of infections across national borders. For ease of access and usability, the data and R scripts have been made available in Zenodo^28^ and released under a Creative Commons Attribution 4.0 International (CC BY 4.0) license, which allows users to freely share, copy, and redistribute the material in any format, as well as adapt and build upon it for any purpose. The availability of the code, including step-by-step instructions, makes it easy to adapt new data to different research needs, facilitating collaborations and the generation of new hypotheses in the field of public health. Future studies could use this repository to simulate different vaccination scenarios, identify regions at highest risk for measles outbreaks, and develop targeted intervention strategies.

### Notes

The views and opinions of the authors expressed herein do not necessarily state or reflect those of the ECDC. The accuracy of the authors’ statistical analysis and the findings they report are not the responsibility of ECDC. ECDC is not responsible for conclusions or opinions drawn from the data provided. ECDC is not responsible for the correctness of the data and for data management, data merging and data collation after provision of the data. ECDC shall not be held liable for improper or incorrect use of the data.

## Data Availability

All data and scripts supporting the results presented in this study have been deposited on Zenodo^28^ for public access.
